# Internal contracting of health services in Cambodia: drivers for change and lessons learned after a decade of external contracting

**DOI:** 10.1186/s12913-018-3165-z

**Published:** 2018-05-22

**Authors:** Sreytouch Vong, Joanna Raven, David Newlands

**Affiliations:** 1 Research Fellow of ReBUILD Consortium, Phnom Penh, Cambodia; 2 Liverpool School of Tropical Medicine, Liverpool, England; 3 Queen Margaret University, Edinburgh, MA Scotland

**Keywords:** Contracting, Special operating agency, Incentive, Cambodia, Implementation challenges

## Abstract

**Background:**

Since the late 1990s, contracting has been employed in Cambodia in an attempt to accelerate rural health system recovery and improve health service delivery. Special Operating Agencies (SOA), a form of ‘internal contracting’, was introduced into selected districts by the Cambodia Ministry of Health in 2009. This study investigates how the SOA model was implemented and identifies effects on service delivery, challenges in operation and lessons learned.

**Methods:**

The study was carried out in four districts, using mixed methods. Key informant interviews were conducted with representatives of donors and the Ministry of Health. In-depth interviews were carried out with managers of SOA and health facilities and health workers from referral hospitals and health centres. Data from the Annual Health Statistic Report 2009–2012 on utilisation of antenatal care, delivery and immunisation were analysed.

**Results:**

There are several challenges with implementation: limited capacity and funding for monitoring the SOA, questionable reliability of the monitoring data, and some facilities face challenges in achieving the targets set in their contracts. There are some positive effects on staff behaviour which include improved punctuality, being on call for 24 h service, and perceived better quality of care, promoted through adherence to work regulations stipulated in the contracts and provision of incentives. However, flexibility in enforcing these regulations in SOA has led to more dual practice, compared to previous contracting schemes. There are reported increases in utilization of services by the general population and the poor although the quantitative findings question the extent to which these increases are attributable to the contracting model.

**Conclusion:**

Capacity in planning and monitoring contracts at different levels in the health system is required. Service delivery will be undermined if effective performance management is not established nor continuously applied. Improvements in the implementation of SOA include: better monitoring by the central and provincial levels; developing incentive schemes that tackle the issues of dual practice; and securing trustworthy baseline data for performance indicators.

## Background

Globally, public management approaches introducing market mechanisms into the public sector have been widely accepted. One such approach is contracting of health services. Since the late 1990s, Cambodia has experienced various forms of contracting in the health sector. From 2009, the contracting model has involved performance incentives and monitoring mechanisms with a greater level of autonomy for health district management. This paper aims to provide a better understanding of the implementation of the contracting model in Cambodia, which can contribute to the redesign of the performance based contracting scheme interventions in Cambodia.

### Contracting in general

Contracting is a process of fulfilling the conditions as in the written agreement by two or more parties [1]. It has been argued that contracting is a potential mechanism to improve health system performance through enhancing accessibility, equity, quality and efficiency of health services by creating collaborations to achieve public health goals [2]. There are several types of contracting; Table 1 provides an overview.

**Table 1:** Overview of different types of contracting

External contracting: Contracting out	An external service provider is engaged through a contract to provide services with maximum control over the resources and how services should be delivered
External contracting: Contracting in	An external service provider is brought in to manage and operate service provision institutions with some control over resources and services arrangements
Internal contracting	Internal contracting is a form of relational contracting whereby responsibility is delegated to peripheral units under the same legal entity e.g. governments contract with public providers i.e. with autonomous institutions which remain under public ownership

There are advantages and disadvantages to all forms of contracting which are dependent upon capacity, environment and culture in which they are implemented. Several elements need to be in place for contracting to be implemented successfully including regulatory mechanisms and their enforcement, alignments of interests, and coherent policies [3]. Contracting is not a magic bullet with several challenges exist, including: increased capacity to plan, manage and monitor the contract, increased costs for contract management and monitoring, and the possible lack of attention to services outside the contract [3–5]. Despite these concerns, many countries have adopted contracting as a tool for improving health service delivery.

### Contracting in fragile and post- conflict countries

Contracting has been implemented in fragile and post-conflict states to increase access to basic primary care services to a large population or geographically difficult accessed population [6–8]. Cambodia adopted this mechanism to recover health service delivery in the country and carried out “contracting experiments” between 1999 and 2003 (see next section for more details). In Haiti, during the late 1990s, supported by USAID, contracts with NGOs that were partially based on performance were reported to improve coverage of preventive care [9]. Since then, this mechanism has been adopted by donors to support other countries’ health sectors such as Afghanistan, the DRC, Guatemala, Liberia and Southern Sudan. In Afghanistan, from 2002, the Ministry of Public Health contracted with 27 NGOs to deliver a basic package of health services in 31 of 34 provinces, and maintained responsibility for service delivery in the remaining 3 provinces [10]. The use of contracting by three major donors increased access to basic health services from 5% in 2002 to an estimated 77% in 2006 [11]. However, despite these improvements in access to services, a survey showed that inequities in access to and use of services as well as costs of care continue with poorer households facing greater barriers [12].

Contracting is not always viewed as the easy option for governments in fragile states. Several issues have been raised: formal contracting can be challenging in settings where political and economic stability cannot be guaranteed; profound cultural and institutional constraints such as social resistance to the involvement of non-state actors; willingness and/or capacity within the non-state sector to enter into contractual arrangements; bidding processes that may erode quality and favour local cronyism; and performance based contracts may rule out informal providers who are often the most important source of health care for poor people [13]. However, in certain settings where reliance on non-state providers is well established and the capacity of the government is weak, contracting out service delivery can represent the only feasible policy option [14].

### Contracting in Cambodia

The civil war left Cambodia with a fractured health system: empty and ruined health facilities and under a hundred health professionals. With the challenges of few resources, an international blockade and continued local armed conflict, the Ministry of Health tried to reconstruct the health system. Cambodia opened up to foreign investments following the UN sponsored national elections and national unification in 1993. International development agencies were interested in supporting the rehabilitation of the health system. Between 1989 and 1995, both the government and donors invested in the health sector, and the first health sector reform (HSR 1) was implemented between 1991 and 1994. The second health sector reform followed which included establishment of the health coverage plan and financing charter and introduction of user fees at public facilities [15].

**Fig. 1:**
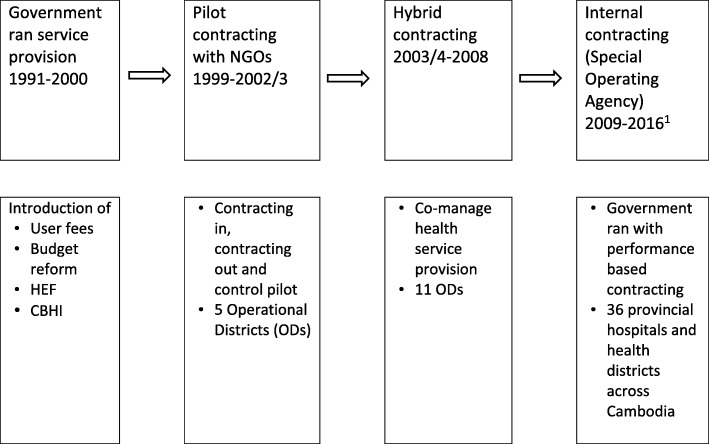
Evolution of contracting health services in Cambodia ^1^SOA status does exist. However, the Service Delivery Grant for SOA is re-designed and scale up across Cambodia, including the non-SOA districts

Since the late 1990s, contracting has been used in Cambodia in an attempt to accelerate the recovery of the rural health system and improve health service delivery. The three major phases of contracting are: 1) the pilot phase of contracting between 1999 and 2002/3; 2) “hybrid contracting”; 3) in 2009, Special Operating Agency (SOA), a form of internal contracting was introduced (Fig. 1).

In phase 1, external contracting operated in five health districts (out of total of 183 in Cambodia), of which two districts used “contracting out” and three used “contracting in”. Improvements in maternal and child health services were reported - increased coverage of health services, more equitable provision of care, and reductions in out of pocket expenditure - but at a cost almost twice as high as standard districts [16]. There were concerns about how this approach could be sustained and whether it was affordable. In phase 2 (2004–8), “hybrid contracting” was introduced in 16 health districts: in 11 health districts international NGOs were contracted to provide management services to the health districts; and the five other districts, were contracted by their provincial departments and received funding from the Belgian Development Agency (BTC). Performance contracts, incentives, monitoring mechanisms and capacity development of local health management were the main features of both models [15, 17]. Increased service utilization, improved transparency and accountability in management, and improved health worker motivation were reported, but no rigorous evaluation was conducted (Keller S et al: Assessment of performance contracting in Kampong Cham Province, Cambodia, unpublished; [18]).

In phase 3, “internal contracting” was adopted. Public service institutions including health districts were transformed into semi-autonomous institutions - SOAs. This brought about a big shift in management arrangements, allowing more autonomy for the districts to manage their resources. Table 2 provides more details about SOA.

**Table 2:** Special Operating Agency

What is a Special Operating Agency?	Special Operating Agency (SOA) is a supply-side oriented mechanism developed from contracting and implemented by the Cambodian government through the use of government staff as contractors to improve the quality of health care services for people, mainly the poor and vulnerable.
How does it operate?	In each Operational District (OD) there are government guidelines for the delivery of the Minimum Package of Activities and Complementary Package of Activities. The SOA is given a degree of autonomy in making decisions about the best use of their human, physical and financial resources to deliver the highest possible quality of services, in the most effective way and to enhance performance and accountability through streamlining administration to be more transparent and responsive to people’s needs. The SOAs are able to hire additional workforce, conduct performance monitoring and evaluation, and provide performance incentives. With the conditions set in the contract and penalties involved in underperformance at SOAs, contract monitoring at these ODs takes place more rigorously and with clear criteria for determining level of performance, a feature not usually seen in standard ODs.
Sources of budget for SOA	A standard OD has two major income streams: the government budget and user fees. SOAs have these plus a package of budget from the Health Sector Support Program in the form of a Service Delivery Grant (SDG). This additional budget (approximately 40% of the total budget managed by ODs) is mainly used for performance monitoring and incentives. The Ministry of Health (MOH) signs a performance agreement with the Provincial Health Department (PHD), and the PHD in turn signs a services delivery management contract with the SOA.
Role of MoH and PHD	The MOH is responsible for timely allocation of funds, provision of policies and guidelines, and enforcement of health legislation, professional ethics and codes of conduct to PHD. The PHD takes responsibilities for providing SOA with financial resources and assistance in human resources and performance management. Under the service delivery management contract, the SOA is responsible for ensuring the management of resources at all facilities. The PHD conducts monitoring of the SOA, usually on joint monitoring visits with the HSSP monitoring team. The HSSP monitoring team includes an external agency.
Extent of SOA operation	Thirty SOAs were established by the end of 2010 and six more SOAs were introduced by 2013.

Several studies have researched the SOA approach. Khim and Annear 2013, assessed this approach as a means for improving the management of district health services and strengthening service delivery in 2011 [19]. They found that it has the potential to improve service delivery, provided that: there is a clear understanding of roles and responsibilities by the contracting parties; implementation of clear rules and procedures; effective management of performance; effective monitoring of the contract; and adequate and timely provision of resources. However, this study was conducted only 1 year after the start of SOA, was conducted in 2 provinces only – with three of the four study sites being in one province, and focused on district, provincial and central level health managers. Khim et al. also looked at the factors driving the changes in design, implementation and scaling up of contracting over the period 1997 to 2015. They found that these changes reflected a broader process of economic and social development in Cambodia [20]. The government has endorsed the SOA contracting approach and will move to national coverage in the foreseeable future. However, contracting governance mechanisms need to be strengthened. Others have investigated utilisation of services through a household survey in 8 districts, four of which were SOAs, and found that utilization of public health facilities for outpatient visits was higher in SOAs than in non-SOAs (World Bank: Cambodia's rural health markets. Human Development Unit & East Asia and Pacific Region: World Bank, unpublished). Basic infrastructure in health centres and health posts (such as the presence of a fridge or sterilizers) was better in SOAs and dual practice was 25% less frequent but vaccination coverage was lower in SOAs for all vaccines. However, the study was not explicitly designed to measure the impact of SOAs nor control for other interventions that may have been implemented in non-SOA districts (e.g., vouchers for maternal health). The authors recommended a more robust monitoring and impact evaluation of the performance of SOAs. A recent study investigated the effects of contracting on utilization of maternal and child health services from 1998 to 2010, but did not include the SOA phase [21].

There has been little research done on how the SOA model has been implemented. There is therefore a need to understand how this contracting model has been implemented over the 5 years and derive lessons learned from implementation to inform any next steps. The study builds on Khim and Annear’s study 2013, but draws upon a wider range of districts and perspectives, as well as exploring a longer period of implementation of the SOA model. The aim of this study is therefore to investigate how the internal contracting model has been implemented, identify challenges, effects on service delivery and provide lessons learned.

## Methods

### Study design and site

A mixed method study was employed. Qualitative methodology, comprising key informant interviews with government and donor representatives and in-depth interviews with managers and health workers, was used to enable direct engagement with the participants and thereby to facilitate an exploration of their views and experiences of contracting. Secondary data from the Annual Health Statistics Report was analysed to identify trends in service utilisation.

The study was conducted in four operational districts (OD). These districts were selected because: they were in different areas of the country; they had a range of experience of previous contracting models; they were currently operating as a SOA; and they had a range of services covered by SOA. In Table 3, information about each district is provided.

**Table 3:** Characteristics of the study districts

District (province)	SOA	Previous contracting	Level of service covered	Geographical area	Population/number of health facilities
Memut (Kampong Cham)	Yes (2009)	• 1999–2002/3 contracting out managed by SCA • 2004–2008 contracting managed by SCA	Primary and secondary care	Lower east plateau bordering Vietnam	135,500 1 referral hospital 10 health centres
Peariang (Prey Veng)	Yes (2009)	• 1999–2002 contracting-in managed by Healthnet International • 2004–2008 contracting managed by Healthnet International	Primary and secondary care	Central south plains	193,500 1 referral hospital 15 health centres
Samrong (Oddor Meanchey)	Yes (2010)	• 2005/2006–2009: performance contract supported by BTC • 2006–2008: PMG	Primary care only	Upper North Mountainous	219,000 1 referral hospital 23 health centres
Bati (Takeo)	Yes (2010)	None	Primary and secondary care	Plain	202,026 1 referral hospital 13 health centres

*SCA* Save the Children, Australia, *BTC* Belgian Development Agency, *PMG* Priority Mission Group

### Qualitative methods

All interviews were conducted by the research team comprising a Cambodian social scientist and research assistant. Topic guides were developed for each type of interview using the government’s SOA manual and published literature.

#### Key informant interviews

Between August and November 2013, 12 key informant interviews were conducted. The key informants included: representatives of the Health Sector Support Project 2 and the Ministry of Health (*n* = 4); representatives from AfD, World Bank, URC, Unicef, UNFPA, BTC, CARE and AusAid (*n* = 8). These interviews explored the key informants’ perceptions and experiences of SOA implementation, challenges and advantages of SOA implementation, and effects on service utilization.

#### In depth interview with managers and health workers

Twenty-seven interviews were conducted with: SOA managers in the Provincial Health Department and the OD; managers of the selected health center and the referral hospital in each district; and health workers (one nurse/ midwife and one doctor from the referral hospital; and one nurse/ midwife from the selected health centre). These interviews explored their views on how SOA has been implemented including the challenges and their coping mechanisms, benefits of SOA, effects of SOA on health system performance including service utilization, and continuation and scaling-up of SOA.

#### Data analysis

All recorded interviews were transcribed verbatim into Khmer. They were then translated into English and assessed for accuracy against the Khmer transcript and the recording. Where recording of interviews was declined (*n* = 2), detailed notes were developed into electronic documents in English. Using the framework approach, the research team (comprising of Cambodia social scientist and research assistant, and UK social scientist) analysed the data. This approach promotes transparent and rigorous analysis [22]. The team read the transcripts and identified emerging themes, which they used to develop a coding framework. The team coded all the transcripts with this framework. The team then developed charts for all themes and created narratives that described similar and divergent perceptions, and developed explanations and find associations between them.

### Secondary quantitative data

The research team collected data on immunization, antenatal care, deliveries by skilled birth attendant and deliveries at health facilities from the Annual Health Statistic report (2009 to 2012) for the four provinces which include the study districts. This data were analysed to describe the trends in coverage for these indicators from 2009 to 2012. We compared coverage between contracting (SOA) and non-contracting (non-SOA) districts in response to the priori hypothesis that SOA districts should perform better than non-SOA districts (and the province average).

## Results

**Fig. 2:**
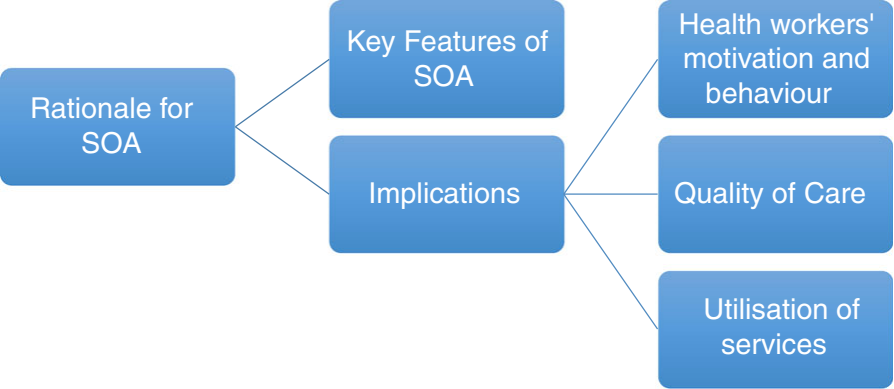
Framework for assessing impact of SOA in Cambodia (adapted from Mills (1998) & Caltadol and Kielmann (2016))

The results are presented using a framework (Fig. 2) which has been adapted from two studies that evaluated contracting schemes to [23, 24]. We have adapted the framework so that it fits with the overall aims of SOA. The components of the adapted framework include the reasons for introduction of contracting, key features of the contract design including how it is implemented, the implications of contacting on health workers’ behaviour, quality of care, and utilization of services.

### Rationale for introduction of SOA

Managers and health workers identified several reasons for the introduction of SOA including regaining national and local ownership of the health system, increased local capacity in managing contract, issues of cost and sustainability, and reducing the conflict of interests in private practice.

#### National and local ownership: Regaining ownership in managing health system

Local managers reported that they had no authority to manage the health services in their district or province, including allocation of budgets or recruiting and firing staff under the previous contracting models with NGOs. Managers and health workers explained that they were employed by the NGOs, and were “pressurized” to work and follow their rules, and felt little ownership of the local health system. They reported more freedom and greater ownership of their health system in the SOA model.

*“Ownership!...there was no ownership at that time because they have managers to manage over us and we were just their staff...We are staff completely employed by NGO…It is normal that civil servant now have more freedom than working with NGO” (Facility chief, Memut).*

#### Local capacity in managing contract

Some managers reported that district and provincial health managers’ capacity to manage contracts and service delivery was developed in districts where contracting with NGOs operated over several years. Working with NGOs helped develop the competencies of local managers to manage the health services independently. This experience facilitated local managers to operate within the SOA. The change to SOA allowed more opportunity to continue building capacity of managers within the current health system.

*“At that time, we still had limited capacities, we are not good at budget management. Before that we only used the government’s budget which was only small amount...when contractor came in, they taught us...” (SOA manager, Memut).*

However, other key informants explained that NGOs operated very independently and did not build the capacity of the local managers to manage their health services.

*“…The problem they found was that working with NGOs didn’t really help in capacity building for government officials…” (KII2, Male, MoH).*

#### Cost and sustainability

Financial sustainability of contracting with NGOs was questioned as the costs of employing NGOs and expatriate staff were high and mainly covered by donor contributions. In some districts, managers and health workers were annoyed with the amount of money being spent on expatriate staff instead of being used for developing local staff.

*“If we continue to hire NGO for contracting, MoH does not have money to pay for that, at the same time staff at lower level …were not happy because NGO took much money, so there is little money left for development” (KII7, Male, Donor).*

#### Private practice

As the base salary of a civil servant in Cambodia is low, private practice becomes a popular means of income substitution for public health workers. During the previous contracting regimes, private practice was strictly banned, creating some tensions between contractors and local managers and providers. In some cases, this resulted in providers leaving their public-sector job. The continue of contracting under this situation put MOH under pressure to tackle with this issue. Therefore, SOA is a more “realistic” contracting approach for the system in Cambodia.

*“Regulation was too strict… they were not allowed to work outside [private practice] but they were provided with incentive. But as usual, incentive was not enough for a decent living. They need it for life, they need it for their living, then they sold drugs and opened their clinic. This was against their contract, and NGO did not agree to allow staff to open their clinics, they fined staff 1 or 2 time then send name of staff to PHD, so some staff just asked to suspend by themselves” (SOA manager, Peariang).*

### Key features of the SOA and their implementation

This section is divided into four themes emerging from the data: selection of districts for SOA; setting and reaching targets in SOA; monitoring of SOA; and incentives provided through SOA.

#### Assessment and selection of SOA

For a district to be a SOA, they must apply to the Council of Ministers and be assessed districts in terms of quality of health facilities within the district, human resource management functions, and the availability of a capacity building agency to support their transition to SOA status. Some key informants perceived that this process leads to the selection of only well performing districts.

*“We already select the better facilities to run SOA, so its nature is already good. In general, if we have 10 students, there must be 2 or 3 outstanding students. Thus, these outstanding ones already have their potential. So does SOA” (KII11, Male, Donor)*

#### Setting and reaching targets

Managers and health workers reported several challenges in achieving the targets that are set. Firstly, managers explained that targets are set using population data. Some managers reported that the government assumes that the population in the district is growing which is not always the case as there is migration in and out of the district, and this can result in overestimation of the population and targets being set too high.

*“Those migrants were not in the target population, so the target increased. However, they came temporarily and went back, so the number decreased, and even more people moved out, for instance, there were 100 people moved in, but 200 moved out. This is what we are worried about” (Manager, Memut).*

Secondly, some managers explained that the baseline data on utilization of services is inaccurate – it was too high and did not reflect the real situation in the district. They perceived that the targets were set using this baseline and were therefore unrealistically high. Managers also explained that the targets should increase every year. This was perceived as an issue by managers in Memut as they already have good performance and reach their high targets and it is very difficult to improve on this.

*“… so far our results have been high and they do not allow us to set them down...they never think that once we reach the peak, how can we reach more?” (Manager, Memut).*

Thirdly, facility managers and health workers reported that they compete with other facilities to attract people to use their services, and therefore reach their targets. Some managers reported that they were able to do this, by visiting homes in the community to promote use of the facilities and provide services such as consultations, antenatal examinations, and vaccinations. However, other managers and health workers found this more difficult as they had fewer staff and so outreach work would leave the facilities understaffed, and transport issues.

*“My place is facing issue of outpatients, because other facilities rarely let their clients pass by to use services here, they have achieved their target already too. While my facility is in the middle of other facilities, so the population around this area is the same, but many health centres absorb the clients.” (Manager, Samrong).*

#### Monitoring of SOA

*Irregular monitoring from the central level:* Routine monitoring of the SOA districts, through visits to the districts and reviewing reports, should be carried out by the Service Delivery Monitoring Group (SDMG) from the central MoH. However, key informants reported that this monitoring was irregular and provided two main reasons. SDMG members are unable to assign enough time to this activity as they have other duties within the MoH; and they receive very little financial incentives for travelling to the provinces for monitoring, which is in sharp contrast to the payments they can receive from private practice in Phnom Penh. Some key informants also reported the limited capacity of some SDMG members to monitor the SOA, which has implications on the quality of monitoring. This central level monitoring should also include donor or NGO representatives to provide an “external” perspective. However, this rarely happened.

*“The SDMG monitoring group from ministry is supposed to conduct monitoring every quarter, but in fact, they only did twice per year. The SDMG consists of 4 members, but sometimes only 1 of them went for monitoring. They have more work, and sometimes they have job outside [private practice], as the per-diem for monitoring is $20/day, if they stay in Phnom Penh and do one operation, they earn $300.” (KII1, Male, MoH).*

*Provincial Health Department (PHD) lack sufficient budget to conduct monitoring:* The PHD should conduct quarterly monitoring visits to the SOA districts. However, there is no specific budget for monitoring the SOA districts and the general monitoring budget is insufficient for the frequency of monitoring visits needed. There are no incentives provided to the PHD managers for monitoring.

*“…In general, PHD doesn’t receive any incentive… Sometimes the PHDs complain… We can see that their work load has increased and they have to work harder, but have no incentive” (KII12, Male, Donor).*

*Monitoring is useful:* Some key informants reported that data can be faked by managers and health care providers so that they are seen as reaching their targets and will receive the financial incentives. They stressed the importance of central and provincial level monitoring that verifies the data. Managers and staff also explained that monitoring visits that include going into the community and verifying that community members actually used the services, encourages staff at health centres to not falsify the facility records.

*“In the past, we didn’t have a monitoring system, so the data provided might not be true or might not be clear*. *After the implementation of SOA, we established proper monitoring system…when our monitoring team went to inspect ANC, we…took the name list and went to inspect. In the morning we were in the health centre and in the afternoon we went to inspect in the village. So they didn’t dare to make false report” (Manager, Bati)*

Monitoring was seen as a way to identify errors and improve the performance of health care providers, such as improving punctuality, and providing good quality care including giving correct treatment according to guidelines and completing documents. Monitoring can also help in clarifying individual health worker role and responsibilities. In the monitoring conducted by the PHD, progress towards the targets in the contract is assessed, and any delays are investigated. The PHD then helps the district managers to find solutions.

*“It is very important. If there is no such evaluation and monitoring, the work cannot be done smoothly and we cannot work effectively. Sometimes we have mistakes, when they come, they will give us advice. Thus, we improve ourselves for better performance…For instance, now we have mistakes, so what should we do to be better for next quarter and further… it is really important” (Manager, Memut).*

#### Incentives from SOA

Managers and health workers perceived that a key benefit of SOA is provision of financial incentives, including bonuses for health workers. Although the health workers perceived that the amount of incentive is not great, it does supplement their salary and incentives from other schemes, and helps with their livelihood.

*“The benefits for staff in health centre is not much, but it is just... to supplement their daily livelihood, it is quite a big amount for them” (Health worker, Peariang).*

*“I’d be happy if SOA continues. Because when having SOA, they provide us some bonus and additional incentive for staff, that’s also good that they have training for us as well” (Health worker, Memut).*

### Implications of SOA

This section includes the following themes: behaviour of health workers; quality of care; and utilization of services.

#### Changes in health workers’ behaviour

*Punctuality:* SOA contracts include punctuality of health staff as one of the criteria for allocation of incentives. Most respondents reported that this has encouraged staff to arrive and leave work on time and according to their duty roster. In previous contracting models, punctuality was also good, but this was due to threat of sanctions such as suspension and withdrawal of payment. Punctuality had improved since the introduction of SOA in districts where contracting had not been implemented. Some respondents explained that SOA rules are less strict than in previous contracting, allowing staff to leave work early or during quiet times so that they can conduct private practice.

*“…SOA is different from non-SOA. For non-SOA, no matter where it is, if you visit there at 3 or 4pm, you will see no one there and they only leave the phone number. Sometimes when people phone staff, they would answer that they are still on the way. So what is the quality? In addition, they also have changed their habits and attitudes and the way they speak to the patients. In the past, they used to get up at 7 or 8 am, but now they change – they have to come to work on time, and be on duty… In the past, they used to come late and treat the patients badly… Now, they have slogan that, ‘Services are to serve people.’” (KII4, Male, MoH).*

*“We have signed the contract with them, so we need to work even though we have a lot of work at home. We need to wait until we finish work at the health centre, then we can do private work at home.” (Facility chief, Peariang).*

*Provision of 24 h service:* Under SOA, the availability of 24 h services has improved. In Bati province, facilities only started providing 24 h services when SOA was introduced. Before this, staff were often absent from the facilities. Managers and health workers reported that the strict rules about attendance at the facilities as well as generating income from user fees promoted staff to be stay at the facilities.

*“There were no permanent 24 hours service here before, like there were services but there was no staff.... in the past, there was only name, no staff...” (Health worker, Bati).*

*Dual practice:* All respondents reported that staff continue to conduct private practice alongside their government work in SOA districts. Dual practice is not prohibited in SOA districts, but is allowed as long as the health worker does it outside of their government working hours and it does not hamper the achievement of the targets set in the SOA contract. However, some health workers carry out private practice during working hours, as they are able to agree with their colleagues to cover their workload. Policy makers explained that the amount of incentives in SOA is not enough to prevent staff from conducting private work. Without generating income from private practice, some specialist staff are likely to leave their government job.

*“If that unit is too strict and does not allow staff to work in private sectors, they would all quit. Sometimes we have to do it differently from the contract, which states that staff have to work 8 hours a day. We even allow the specialists to work for 4 hours a day in order to avoid their resignation. For example, if we do not allow a surgeon to operate in any other clinics besides the state hospitals, they will all quit work.” (KII3, Male, MoH).*

#### Quality of care

Managers and health workers in study areas reported that quality of care had improved following the introduction of SOA. Staff are more careful about how they assess and treat clients. They explained that they now carry out more comprehensive assessments of the patients according to guidelines, clearly record the assessment findings and diagnosis, see one patient at a time, and only prescribe for patients who attend the facility and not prescribe for people who remain at home. They also explained that staff were friendlier to clients, and showed more kindness and consideration. These improvements had started during previous contracting regimes, as health workers became accustomed to following standards and procedures, but the monitoring and provision of incentives in SOA have reinforced this behaviour.

*“Before, in consultation we just asked a few things, and then just wrote the prescription. But when having SOA, we have to measure blood pressure, measure body temperature. Later, we note on the paper. For example, if a patient is having fever, we just ask them to do blood testing before we can provide them prescription…before SOA, we just often did short cut ways [for the treatment]... there was no monitoring from upper level, there were no incentives, [we] were just lazy too, that’s why…” (Health worker, Samrong).*

*“[diagnosis] has changed, in the past they all tried to squeeze in…like ten patients tried to get examination at one time. Today no! One person at a time, with number order” (Health worker, Bati).*

*“Staff change the way they talk to patients because before we had low sense of responsibility..., in short it was because of little money [incentive]. Our work is better than before. Having the incentive from SOA, makes us work better.” (Health worker, Samrong).*

#### Utilization of services

**Fig. 3:**
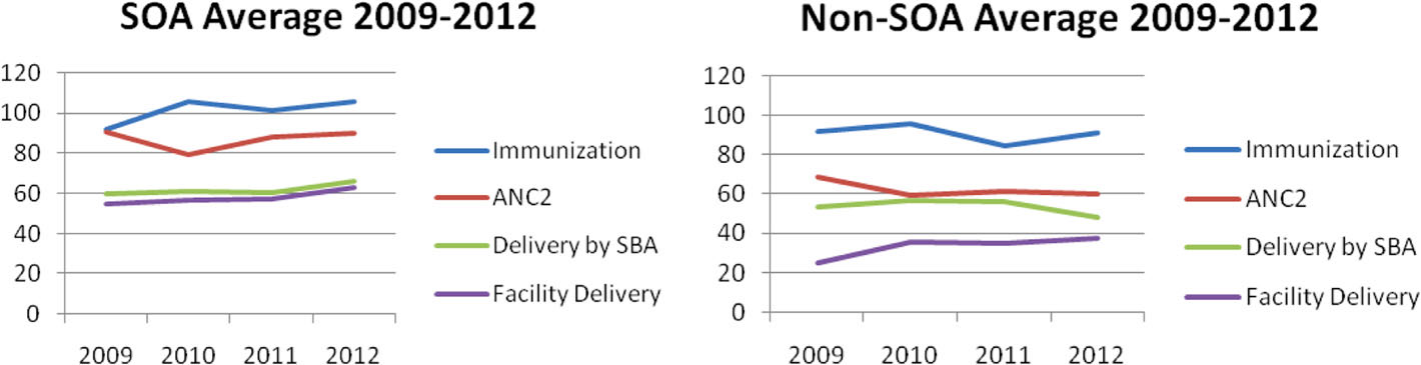
Average service coverage in SOA and non-SOA districts (2009–2012)

In 2009, when SOA model was introduced, the average coverage rate for immunization was similar in the 13 SOA districts to the average rate in the 10 non-SOA districts., However, average coverage rates of deliveries by Skilled Birth Attendant (SBA), antenatal care and deliveries in a health facility were higher in the SOA districts than the non-SOA districts (Fig. 3). Over the period of 2009 to 2012, SOA districts performed better on average than non-SOA districts on antenatal care and immunization.

In the SOA districts, there was a 10% increase in deliveries by SBA from 2009 to 2012. In the non-SOA districts, there was a 10% fall over the same period. However, an increase of 13% from 2009 to 2012 is evident if one district (out of 10 non-SOA districts), which has scarcely plausible data, is excluded. Non-SOA districts also realized a much greater increase in health facility deliveries than SOA districts.

Managers and health workers reported that since the introduction of SOA, utilization of services has increased. The major reasons for these increases are improved public trust in services provided and the availability of 24 h services (Table 4).

*“They just know that our staff work here regularly so that they come to use services here...because when they come they always meet our staff so they just like to come more. But before when they came, they didn’t meet our staff so they didn’t like to come any more” (Health worker, Samrong).*

**Table 4:** Reasons for increases in service utilization in SOA districts

Key informants	Health managers and providers
Public have more trust in the SOA facilities	Improved public trust in health facilities – provide better quality of care (improved staff attitude and better treatment)
Facilities are now open for 24 h per day	Staff being available 24 h per day
Staff are more punctual and stay at facilities because of incentives attached to punctuality and availability of services	Outreach programmes have increased community awareness of the availability of and need for services
Staff have received more training since being in SOA and this has improved the services that they deliver	Clear contracts with targets for provision of services, incentives and monitoring in the SOA scheme
	Low service fees because of HEF and CBHI

Managers and health workers also reported increases in service use by the poor. They identified three main reasons for these increases: Health Equity Funds (HEF), a funding mechanism that gives vulnerable and poor populations access to health services, are available in all SOA districts; SOA contracts specify quality of care including communication with clients and this has encouraged staff to behave well towards all clients irrespective of socio-economic status; and facilities are open 24 h, so that the poor can access services at any time.

## Discussion

### Summary of key findings

This paper explores the challenges in implementing the SOA approach, the effects on service delivery and identifies any lessons learned from implementation to inform next steps. There are several key findings from this study. There are issues with the capacity and funding for monitoring the SOA, as well as questions marks about the reliability of the data. Some facilities face challenges in achieving the targets set in their contracts. There are some positive effects on staff behaviour which include being more punctual at facility, being on call for 24 h service, improved communication with clients, and perceived better quality of care, promoted through adherence to work regulations stipulated in the contracts and provision of incentives. Compared to former contracting schemes, dual practice has increased as there is some flexibility in enforcing the contract regulations in SOA. Utilization of services has increased according to the managers and health workers largely because of increased community trust in health facilities and services being provided throughout the 24 h period. Reported increased service utilisation by the poor was attributed to the HEF being available in all SOA districts and improved attitudes of health workers. However, the secondary data provide a different picture about utilisation of services. Emerging from the findings are three important areas for discussion: achieving targets and the quality of data; issues with monitoring; and quality of care.

#### Achieving targets and the quality of data

Some facilities found it difficult to achieve the targets set in the contracts. The findings suggest several issues. The population data used to calculate the targets may be inaccurate. This is not unique to the Cambodian context, as many countries face challenges in acquiring accurate population data [25]. The service utilization data used as baseline for targets are also suspected to be inaccurate. Too high baseline figures for service utilization, make it difficult for increases to be demonstrated. Definition of denominators and a lack of complete data for indicators were similar challenges identified in a recent study [19].

This raises the issue of the quality of the data for not only baseline assessment and target setting but also monitoring performance and assessing the impact of SOA on service utilization. The secondary data suggests SOA districts performed better than non-SOA districts for antenatal care and immunisation. However, the opposite was true for health facility deliveries and (probably) also for SBA deliveries. There are several difficulties in interpreting the secondary data. The selection bias of districts as districts were selected for SOA as they were already well-performing. There are more resources available to SOA districts than to non-SOA districts. Other interventions apart from the contracting process have been implemented in the provinces such as the Health Equity Fund, the Government Midwifery Scheme, the Reproductive Health Voucher Scheme and the Community Base Health Insurance Scheme. Applying uniform national or provincial ratios to district populations to estimate specific populations e.g. the number of pregnant women or children under 1 year can be problematic. If the actual figures are greater than the assumed figure, then the real coverage rate may be lower. There were also many examples of scarcely believable values or changes in the data. For example, in one district the rate of SBA delivery fell from 88 to 33% over 3 years. Although possible reasons for this may also lie in new facilities drawing clients away from this district.

Creating competition amongst health care facilities can drive increases in utilization and quality of care [26]. Facilities work hard to attract people to use their services through improved quality of care and outreach work. However, some facility managers and health workers found this difficult. Reluctance to compete with their colleagues in other facilities for clients, inability or unwillingness to increase outreach work and improve quality of care, or sheer overload of work may be factors influencing their approach to competition. Support from OD and PHD managers is needed to identify how facilities can attract more clients within their context.

#### Issues with monitoring

Monitoring the performance of the health services is an important aspect of contract management [5]. However, in this study there are weaknesses in monitoring from central and provincial level health departments – infrequent visits, limited capacity to conduct thorough monitoring, and inadequate external review. These are not new findings, but have continued throughout the implementation of the SOA: monitoring had not been included or budgeted for in their annual operation plans; a lack of clarity in roles and responsibilities of the contracting parties; and the overlapping roles of the SOA and monitoring groups (World Bank: Cambodia's rural health markets. Human Development Unit & East Asia and Pacific Region: World Bank, unpublished; [20]). There are several reasons behind the issues of monitoring. Monitoring is not incentivized or linked with performance contract especially the contract between MOH and PHD, making them reluctant to perform their monitoring roles. This reluctance is aggravated as other staff in the OD and health care workers receive incentives from the SOA scheme. One other revolves around the perceived usefulness of monitoring: managers could only withhold incentives when performance is poor and some staff may be willing to go without them as they were able to generate income elsewhere. Although one of the driving forces for introducing the SOA was national and local ownership of the health system, this may have not trickled down to the district level, with the perception that the central level still directs and makes decision, including with monitoring.

Monitoring activities should be included in the annual operation plan so that they have sufficient budget and are prioritized (World Bank: Cambodia's rural health markets. Human Development Unit & East Asia and Pacific Region: World Bank, unpublished). Independent performance monitoring has been recommended by a recent study in Cambodia (World Bank: Cambodia's rural health markets. Human Development Unit & East Asia and Pacific Region: World Bank, unpublished). This study also suggests that SOA monitoring could be improved through the consistent use of a third party.

Weak monitoring is not exclusive to the Cambodian context. There is evidence from other countries that there is weak capacity of the state in monitoring the performance of contracting [5]. Investment in the development of robust monitoring mechanisms in terms of capacity to monitor and adequate funding is vital if contracting is to be successful in improving access, utilization and quality of services.

#### Quality of care and dual practice

The study suggests that within the SOA approach, there is some enforcement of regulations such as wearing uniform, following guidelines and providing 24 h services. There is also evidence of some flexibility in working arrangements, allowing for less strict working hours, and some private practice. Indeed, relaxation of the ban on private practice was seen as one of the driving forces behind the introduction of SOA. This may be a pragmatic solution followed by managers who consider that the government salary and financial incentives (including the SOA incentive) are not enough to attract and retain staff, and that private practice can generate important income. There is some debate in the literature about the implications of dual practice on service coverage. There is concern that it can reduce quality of care and accessibility to users of the public system [27]. Others suggest that opportunities for public sector health workers to practice in the private sector keeps them in the public sector, who would otherwise migrate overseas, move into full time private practice or into other professions [28]. The implications of dual practice are context specific, dependent on the labour market, regulatory authority, and demand for services [29]. In our study setting there are important issues with allowing dual practice to consider. Service users may have to wait for the health workers to arrive at the health facility, causing delays in receiving treatment or referral, which could potentially be life threatening. User dissatisfaction, reduced willingness to use the facility again or promote usage of services by their family or community are all repercussions of delays in being seen at health facilities. In addition, dual practice raises equity issues. Poor patients are more likely to use public facilities where the Health Equity Fund will support their costs, and so they are the ones who will suffer most from the delays in receiving treatment.

But how do you deal with dual practice? This is challenging as it is a well-established practice embedded in health organizations. There are a number of approaches to take: a review of health worker salary and incentives across the cadres in order to understand the drivers of health worker behavior - income and the perception of a fair distribution of incentives is associated with higher job motivation scores [30]; developing a holistic incentive package that includes non-financial incentives such as preferential access to training, provision of accommodation, clinical mentoring, and improved transport and working conditions [31]; together with building on the intrinsic motivation of staff to serve their community [30].

The study has several limitations. We did not include user perspectives on changes in utilization and quality of services. Although we have perceptions on changes in quality of care from health workers and managers, we did not focus on the effects of SOA on quality of care. A study of quality of care using both quantitative and qualitative methodology would be important. We did not investigate in-depth the issues of dual practice in the current study. Further study should focus on effects of dual practice and its implications on the performance based contracting. We were unable to systematically identify the role of contracting as such in the observed changes. There are problems with attribution with regard to the operation of the Health Equity Fund in all SOA districts and the fact that SOA districts received greater funding than non-SOA ones.

## Conclusion

Over the last 8 years, the SOA model of contracting has been implemented in 36 provincial hospitals and health districts in Cambodia. The SOA model can improve health worker performance: health workers adhere to work regulations and are rewarded with incentives as specified in the contracts in SOA. However, managing contracts in SOA is a complex process requiring capacity in planning and monitoring contracts at different levels in the health system. Service delivery will be undermined if effective performance management is not established nor applied. If the SOA contracting system is to continue, then improvements in the implementation of SOA are needed: improved monitoring by the central and provincial levels; developing incentive schemes that tackle the issues of dual practice; and securing trustworthy baseline data for performance indicators.

## Abbreviations

HEF: Health equity fund
IDI: In depth interview
KII: Key informant interview
MoH: Ministry of health
OD: Operational district
PHD: Provincial health department
SOA: Special operating agency
SDG: Service Delivery Grant
